# The Link Between Human Alkyladenine DNA Glycosylase and Cancer Development

**DOI:** 10.3390/ijms26157647

**Published:** 2025-08-07

**Authors:** Olga A. Kladova, Aleksandra A. Kuznetsova

**Affiliations:** Institute of Chemical Biology and Fundamental Medicine, Siberian Branch of Russian Academy of Sciences, 630090 Novosibirsk, Russia

**Keywords:** DNA repair, alkyladenine DNA glycosylase, single-nucleotide polymorphism, enzymatic activity, DNA repair coordination

## Abstract

Alkyladenine DNA glycosylase (AAG) is a critical enzyme in the base excision repair (BER) pathway, responsible for removing a broad spectrum of alkylated DNA lesions. While AAG maintains genomic stability, dysregulated activity has been implicated in cancer development, drug resistance, and neurodegenerative diseases. This review synthesizes the current knowledge on AAG’s structure, catalytic mechanism, and polymorphic variants, highlighting their potential roles in disease pathogenesis. A comprehensive bioinformatics analysis of over 370 AAG single-nucleotide polymorphisms (SNPs) is presented, identifying ~40% as high-risk variants likely to impair enzymatic function. Notably, 151 SNPs were predicted to be damaging by multiple algorithms, including substitutions at catalytic residues and non-conserved sites with unknown functional consequences. Analysis of cancer databases (COSMIC, cBioPortal, NCBI) revealed 93 tumor-associated AAG variants, with 18 classified as high-impact mutations. This work underscores the need for mechanistic studies of AAG variants using structural biology, cellular models, and clinical correlation analyses. Deciphering AAG’s polymorphic landscape may unlock personalized strategies for cancer prevention and treatment.

## 1. Introduction

The influence of various environmental factors leads to the damage of cellular DNA. The resulting DNA lesions can lead to the occurrence of mutations in the genome and, as a consequence, to the disruption of the genomic integrity. To preserve the stability of the genome in living organisms, there are several mechanisms of DNA repair [1,2,3,4,5]. The base excision repair (BER) pathway is responsible for removing non-bulky DNA lesions; it is an elaborate system that repairs a wide range of damaged heterocyclic DNA bases [6,7,8,9,10]. The first participants in this process are DNA glycosylases, which identify, recognize and remove the damaged DNA base [11,12,13,14,15,16,17,18,19,20]. The resulting apurinic/apyrimidinic site (AP site) can then be hydrolyzed by the human AP endonuclease 1 (APE1) [21]. The next step involves DNA polymerase β (Polβ), whose main function is to fill the gap formed in DNA after the action of DNA glycosylases and APE1 [22,23,24]. The final step in repairing the original DNA sequence is the ligation of the single-strand break by DNA ligase I or the XRCC1/LigIII complex [25,26,27,28,29,30].

Alkylated DNA lesions can arise from both endogenous sources (such as S-adenosylmethionine) and exogenous agents such as environmental toxins or anticancer chemotherapy drugs [31,32]. Most alkylating drugs are methylating agents that form adducts at the N and O atoms in heterocyclic bases. These lesions are toxic for cells because they can block DNA replication [33]. The repair of DNA lesions caused by alkylating agents is efficiently performed by the BER pathway. Alkyladenine DNA glycosylase AAG (also called methylpurine DNA glycosylase MPG, or ANPG) is the major BER enzyme that recognizes and removes a wide range of modified alkylated DNA heterocyclic bases [32,34]. This DNA glycosylase is a monofunctional enzyme and hydrolyzes the N-glycosidic bond of the lesion containing nucleotide, resulting in the formation of an AP site, which is also cytotoxic and mutagenic [35,36]. Changes in AAG activity due to the expression of functionally deficient enzyme variants can lead to the accumulation of unrepaired DNA damage. One of the reasons for the expression of enzyme variants with altered properties may be the consequence of the substitution of a single nucleotide in the gene encoding the protein—single-nucleotide polymorphisms (SNP). It is currently known that natural SNPs are widespread in the human population, and variants of DNA repair enzymes are often associated with the occurrence of gene-associated diseases [37,38,39]. In this paper, we analyzed the possible influence of natural SNPs on the structure and functions of AAG. Based on the analysis, we identified the AAG polymorphic variants that have a high potential to negatively affect the enzyme’s functioning. SNPs in DNA repair enzymes like AAG are critical to study for several reasons, spanning fundamental biology, disease mechanisms, and clinical applications. SNPs in AAG may impair DNA repair, leading to accumulation of mutagenic lesions and genomic instability—a hallmark of cancer. The impaired repair of oxidative lesions due to AAG SNPs could contribute to neuronal damage, similar to 8-oxoguanine DNA glycosylase (OGG1) variants in Alzheimer’s disease [40]. The interindividual variability in disease could be correlated with AAG polymorphisms activity and could explain why some individuals develop cancers despite similar exposures to, for instance, tobacco carcinogens. Moreover, AAG activity can influence chemosensitivity. Tumors with hyperactive variants may resist alkylating agents, while hypoactive variants could enhance drug efficacy. SNPs in AAG can be functional modifiers of DNA repair capacity with direct links to disease etiology, therapy response, and population health. Decoding AAG SNPs is a gateway to personalized cancer prevention, targeted therapies, and a deeper understanding of genomic instability.

## 2. Structure and Catalytic Mechanism of AAG

DNA glycosylases specific for alkylated DNA lesions have been found in all living organisms from archaea and bacteria to higher eukaryotes. The structural arrangement of alkylpurine DNA glycosylases allows these enzymes to be divided into three structural families: the AAG family, including human alkyladenine DNA glycosylase, the ALK family, including prokaryotic DNA glycosylases AlkC and AlkD, and the HhH-GPD family, containing all other alkyladenine DNA glycosylases [41,42,43,44]. Notably, AAG lacks the helix–hairpin–helix DNA-binding motif that is characteristic of many DNA glycosylases. Thus, AAG and other homologous mammalian alkyladenine DNA glycosylases belong to a separate structural group [44,45,46,47]. Despite the different architecture, AAG and the HhH-GPD family enzymes have structurally similar active sites containing functionally homologous amino acid residues interacting with the everted nitrogenous base, whereas enzymes of the ALK family differ significantly both in structure and in the mechanism of damage recognition [48].

**Figure 1:**
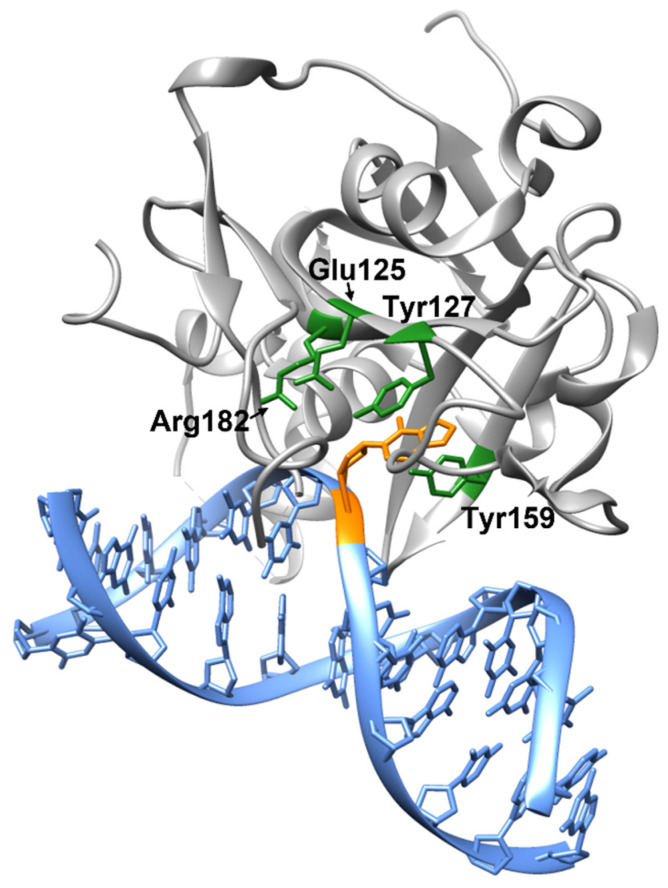
Structure of the AAG enzyme in complex with DNA containing 3,N^4^-ethenocytosine residue (PDB ID:3QI5). The enzyme is gray, DNA is blue, and the 3,N^4^-ethenocytosine residue is orange. The amino acid residues involved in catalysis and coordination of the damaged DNA base are highlighted in green. Glu125 and Arg182 participate in the coordination and deprotonation of the water molecule necessary for hydrolysis of the N-glycosidic bond. Tyr127 and Tyr159 contribute to the correct orientation of the damaged nucleotide in the enzyme active site.

The AAG is a monosubunit protein consisting of 298 amino acid residues and possessing a mixed α/β structure (Figure 1). The *mpg* gene encoding human AAG is located on the short arm of chromosome 16 and consists of five exons, two of which (1a and 1b) are localized in the promoter region. As a result of post-transcriptional modification, several AAG isoforms with different transcript lengths can be formed [49,50,51].

AAG contains an extended unstructured N-terminus, the role of which remains unclear to this day. This enzyme region may potentially participate in interactions with other proteins. For example, it was shown that AAG participates in a complex with actively transcribing RNA polymerase II [52], probably due to the direct interaction of the unstructured N-terminal region of AAG with the ELP1 subunit of the Elongator transcription complex.

The AAG enzyme belongs to the class of hydrolases and the hydrolysis of the N-glycosidic bond of the damaged heterocyclic base occurs through the activation of a water molecule. The amino acid residue Glu125 deprotonates a water molecule, which then attacks the C1′ atom of deoxyribose, followed by damaged base release. The amino acid residue Arg182 also participates in the coordination of the water molecule. The amino acid residues Tyr127 and Tyr159 contribute to the correct orientation of the damaged nucleotide in the enzyme active site. While Tyr127 forms a stacking interaction with the base plane, the Tyr159 is oriented perpendicular to it [44].

**Figure 2:**
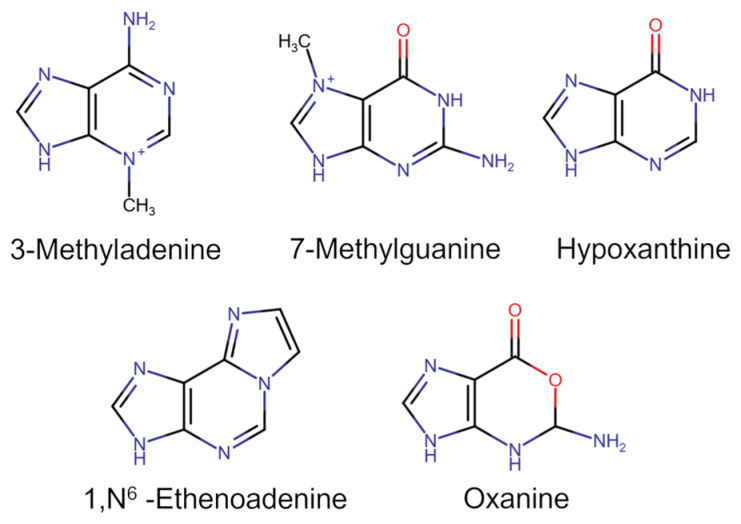
Cleavable AAG substrates.

Human AAG is responsible for the recognition and removal of alkylated purine bases such as 3-methyladenine, 7-methylguanine, or 1,N^6^-ethenoadenine (Figure 2). 7-Methylguanine comprises more than 70% of the total alkylated products following treatment of DNA with monofunctional methylating agents, while 3-methyladenine accounts for about 10% [53,54]. Although 7-methylguanine itself is not cytotoxic or mutagenic, it can undergo spontaneous depurination with the formation of toxic and mutagenic AP sites. In contrast, 3-methyladenine can block replication [55]. 1,N^6^-ethenoadenine is an endogenous DNA lesion formed during the action of lipid peroxidation products and aldehydes on DNA [56,57]. AAG is also capable of removing the hypoxanthine residues from DNA, formed as a result of oxidative deamination of adenosine [58,59], as well as removing oxanine residues from double-stranded and single-stranded DNA, formed as a result of guanosine oxidation [60]. Thus, AAG has broad substrate specificity [49], and a decrease in its activity can lead to the formation of unrepaired alkylated/oxidized DNA bases.

It should be noted that the full-length AAG has different catalytic activity compared to the truncated enzyme lacking the unstructured N-terminal tail, which is most often used in experimental studies. For example, the full-length enzyme can cleave single-stranded DNA containing a uridine residue [56]. The presence of the unstructured N-terminal tail of AAG in cells apparently modulates the enzyme activity. It has also been shown that unlike the truncated enzyme, wild-type AAG does not cleave DNA substrates containing 1-methyladenine, 3-methylthymine, 3-methylcytosine, 3-methyluracil, 3-ethyluracil, and 3,N^4^-ethenocytosine. However, AAG can still bind the lesions that cannot be removed, which leads to unproductive binding [56]. On the one hand, such unproductive binding can inhibit the repair of these lesions by direct oxidative demethylation enzymes [61]; on the other hand, it can interfere with the interaction of AAG with its own substrates [62].

## 3. Polymorphic Variants of AAG and Other DNA Repair Enzymes

To date, a large number of studies have demonstrated the link between the occurrence of cancer and neurodegenerative diseases and the expression of mutant variants of DNA repair enzymes. One mechanism by which such mutant variants can arise is through single-nucleotide substitutions, also called single-nucleotide polymorphisms (SNPs). The prevalence of SNPs and resulting amino acid substitutions in base excision repair enzymes is widespread in the human population [63,64,65]. For many polymorphic variants of enzymes, their altered activity and association with cancer and neurodegenerative diseases have been demonstrated [66]. Studies showed that the polymorphic variant of DNA polymerase β (Polβ) Lys167Ile was found in patients with esophageal carcinoma [67]. It was shown that Lys167Ile amino acid substitution significantly reduces enzyme activity. The polymorphic variant of Polβ Glu288Lys is shown to be associated with rectal cancer, and this mutant form increased the mutation frequency threefold compared to the wild-type enzyme [68]. Polymorphic variants of OGG1 Ala53Thr and Ala288Val have reduced enzymatic activity and were found in patients with Alzheimer’s disease [40]. The low-activity variant of DNA glycosylase NEIL1 Gly83Asp has been associated with an increased risk of cancer [69]. The polymorphic variant of AP endonuclease 1 (APE1) Asp148Glu has been associated with the development of breast cancer, gastrointestinal tract cancer and a moderate risk of lung cancer [70,71,72]. Another polymorphic variant of APE1, Arg237Cys, which has reduced AP-lyase and exonuclease activity, has been associated with amyotrophic lateral sclerosis [73]. The relationship between polymorphisms of genes encoding BER proteins and the etiology of some human diseases remains an open and attractive area for research.

Despite extensive data on polymorphic variants of other DNA repair enzymes, similar studies of AAG mutations are lacking. Several SNPs of the AAG enzyme have been identified in human populations through genetic analysis, but for most variants, their clinical and functional significance remains unclear. Adhikari et al. has characterized polymorphic variants of AAG containing amino acid substitutions at positions K22Q, P64L, Y71H, Q93R, R120C, R141Q, A258V and A298S [74]. These studies showed a neutral effect for mutant variants K22Q, P64L, Y71H, Q93R, A258V and A298S, with enzymatic activity toward hypoxanthine and 1,N6-ethenoadenine containing DNA similar to that of wild-type AAG. Interestingly, the polymorphic variants R120C and R141Q showed decreased activity compared to the wild-type enzyme. Mouse embryonic fibroblast (MEF) cells expressing these AAG mutants also showed increased levels of unrepaired DNA bases. For the intron variant of the *mpg* gene rs2858056, an association with rheumatoid arthritis risk was demonstrated [75,76]. Thus, the functional characterization of natural polymorphic variants of AAG remains an area requiring more detailed analysis.

Giving the limited data on the enzymatic activity of polymorphic variants of the AAG enzyme, this work presents a systematic study of AAG mutants to characterize the possible consequences of amino acid substitutions in this enzyme.

## 4. Analysis of SNP Variants of AAG

There are many bioinformatic approaches that currently allow for prediction of the effect of SNP on protein function. This paper focuses on the analysis of known AAG natural polymorphisms from the NCBI dbSNP database (http://www.ncbi.nlm.nih.gov/snp, accessed on 1 February 2025) to evaluate the possible effects of SNPs on its functions. According to the NCBI dbSNP database, the *mpg* gene contains more than 4600 different single-nucleotide substitutions, a significant proportion of which are located in non-coding intron regions. SNPs leading to a change in the class of amino acid residue (non-synonymous or missense mutations) located in the coding exon region were selected. SNPs resulting in amino acid substitutions were analyzed using six software applications: SIFT (sorting intolerant from tolerant [77,78]), PolyPhen-2 (polymorphism phenotyping v.2 [79,80]), CADD (combined annotation dependent depletion [81,82]), Mutation taster [83,84], MetaRNN [85] and Provean (protein variation effect analyzer [86,87]) to identify the possible influence on enzyme activity. The applications use different algorithms for predicting the possible impact of amino acid substitutions and, thus, using several applications allowed us to increase the accuracy of the predictions and obtain a ranked list of SNPs. Analysis using only one predictive approach may be insufficient since SNPs with predictions near threshold values may be erroneously classified. Also, it should be emphasized that using only a prognostic approach to describe the impact of SNPs is not enough. Experimental verification is necessary to confirm the results obtained using bioinformatic methods.

SNPs were classified as follows (Table S1):

- High negative impact: negative prediction in ≥5 of 6 programs.
- Medium impact: negative prediction in 3–4 programs.
- Low impact: negative prediction in ≤2 programs.

The prediction programs rely on X-ray structural data, protein sequence homology, and physical properties of amino acid residues; so, the results obtained for amino acid residues located in the unstructured N-terminus may not accurately reflect the actual impact of the SNP.

After screening, 151 polymorphic variants were predicted to have damaging effects in at least five of the six programs used. The selected amino acid substitutions were distributed throughout the protein sequence, except in the unstructured N-terminal region. This approach also identified substitutions of functionally significant amino acid residues, including catalytically important Glu125Lys, Tyr127Cys and Tyr159Ser. For the polymorphic variants Arg120Cys and Arg141Gln, which were previously characterized [74], analysis revealed an average probability of a detrimental effect on the enzyme function based on predictions from 4 of 6 and 3 of 6 programs, respectively. For the mutant forms K22Q, P64L, Y71H, Q93R, A258V and A298S, the predicted effect was neutral, consistent with previous findings showing comparable activity to that of wild-type AAG [74]. However, most of the selected polymorphic variants do not involve catalytically significant amino acid residues, and, consequently, their functional significance remains unclear.

## 5. The Role of AAG in the Development of Oncological Diseases

The role of DNA repair enzymes in the development of cancer is still an active area for scientific research [66,88]. Increased expression of mammalian enzymes specific to methylated damaged sites in DNA may differentially influence the sensitivity of cells to alkylating agents [89,90]. Aberrant activity has been shown for many enzymes in various tumor types. Although AAG functions to remove damaged DNA bases, studies have shown that elevated enzyme levels can promote mutagenesis and genetic instability through excessive formation of apurinic/apyrimidinic sites [91,92,93].

It was shown that the mRNA and protein expression levels of AAG were higher in breast cancer samples compared to normal breast epithelium cells [94]. Nuclear staining showed that, in tumor cells, AAG can be located outside the nucleus. Such redistribution of AAG localization can be associated with the features of post-translational modifications in tumor cells, and lead to a decrease in DNA repair mechanisms in the nucleus despite higher expression levels of AAG protein [94].

The influence of AAG and OGG1 activity on the development of lung cancer in smoking and non-smoking patients was investigated [95]. It was shown that high AAG enzyme activity is associated with lung cancer risk, which is opposite to the effect of OGG1. At the same time, no relationship was found between smoking and high AAG activity.

The level of AAG expression was shown to be higher in glioma cells than in healthy brain tissue [96]. Using an immunohistochemistry assay, it was shown that the positive staining for AAG was mainly observed in the nuclei of tumor cells in glioma tissues, whereas its immunoreactivities in non-neoplastic brain sections ranged from undetectable to low. Liu et al. showed that the survival rate of patients with positive AAG staining was lower than those without. Further analysis showed a significantly worse overall survival for patients whose tumors had high AAG levels. This study has demonstrated the prognostic value of AAG levels in human cancers and the potential of high AAG protein levels as a marker of poor prognosis for patients with gliomas.

AAG confers resistance to temozolomide, a primary option for glioma treatment [97,98]. Furthermore, ulcerative colitis—a chronic inflammatory condition linked to colorectal cancer risk—demonstrates increased AAG activity [99]. In mouse models, AAG serves as a key suppressor of inflammation-induced colon cancer, where repair of methylation-induced DNA damage is critical for tumor suppression [100]. *Mpg* knockout mice infected with *H. pylori* exhibited greater susceptibility to preneoplastic gastric lesions [100].

AAG activity varies across the population, and alterations may contribute to cancer development/progression [95,101]. These variations may stem from AAG polymorphisms. While polymorphic variants have been identified in tumors, their functional impacts remain systematically uncharacterized.

Analysis of COSMIC (https://cancer.sanger.ac.uk/cosmic, accessed on 10 February 2025), cBioPortal (https://www.cbioportal.org, accessed on 10 February 2025) and National Cancer Institute (https://portal.gdc.cancer.gov, accessed on 10 February 2025) somatic mutation databases revealed numerous AAG missense mutations in tumors (Table S2). The AAG mutant variants were sorted by cancer type (Table 1). Polymorphic variants of the AAG enzyme were found in tumor tissues of different origins, which may indicate a general role of AAG in the development of cancer. Notably, variants R118Q, P130S, M164I, D175N, L180F and V264A were detected in brain cancers, where AAG’s therapeutic relevance was established [96,97,98].

We analyzed 93 SNP variants of AAG found in cancer samples present in databases (Table S2) and compared the obtained variants with the data in Table S1. Among them, 18 amino acid substitutions correspond to a high degree of negative impact, 7 substitutions correspond to a medium one, and 20 substitutions correspond to a low probability of negative impact on AAG function. Particularly noteworthy are variants that both predict functional impairment and occur in tumors (Table 2). These likely exhibit altered enzymatic properties affecting DNA interaction kinetics.

**Figure 3:**
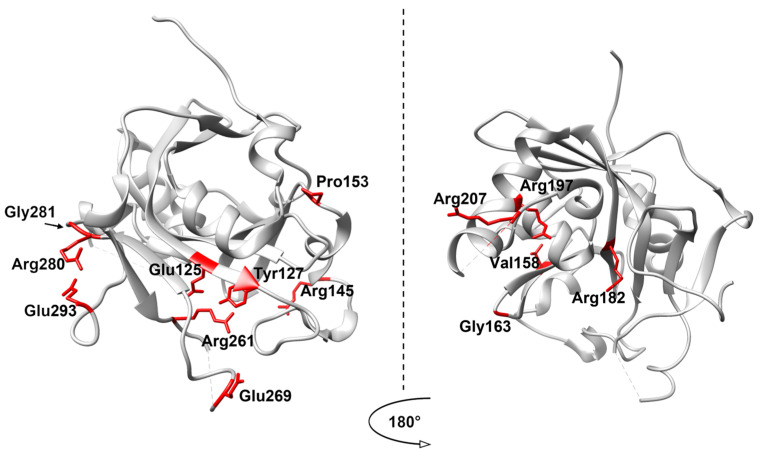
The location of amino acid residues in the AAG molecule with a predicted high negative impact. The protein globule is shown in gray, the highlighted amino acid residues are shown in red. PDB ID:3QI5.

All amino acid substitutions are distributed throughout the protein globule (Figure 3). Also, we identified several conservative amino acid residues in this list: Glu125, Tyr127, Arg145, Pro153, Val158, Gly163 and Arg182. The catalytic residues Tyr127, Arg182, and Glu125 clearly explain their high-risk predictions. For other variants, functional consequences require further experimental confirmation.

## 6. Discussion

The alkyladenine DNA glycosylase AAG is responsible for the removal of a wide range of structurally different alkylated DNA bases. While AAG maintains genetic stability, elevated enzyme activity in certain tumor types confers resistance to chemotherapeutic drugs. The future investigation of AAG and its natural mutant variants can proceed in several directions, with potential implications for cancer biology, therapeutic development, and personalized medicine. The mechanistic insights into AAG dysregulation should be investigated. High-resolution structural studies (cryo-EM or X-ray crystallography) of AAG SNP variants could elucidate how specific SNPs alter enzyme–substrate interactions, catalytic efficiency or DNA-binding affinity. Systematic investigation of post-translational modifications (phosphorylation, acetylation) in AAG’s unstructured N-terminus may reveal regulatory mechanisms influencing its activity or localization in cancer cells. The functional characterization of predicted high-risk SNP variants should include in vitro assays and cellular model assays. Particular attention may be paid to establishing the role of SNPs leading to the substitution of non-conservative amino acid residues. The functional significance of most of these amino acid substitutions remains unclear and requires additional studies to confirm the predicted high negative impact on AAG function. Also, the examination of how AAG variants affect coordination with other DNA repair enzymes (for example, APE1, OGG1, Polβ, or the XRCC1/LigIII complex) can additionally provide insights into the dysregulation of DNA repair mechanisms.

Research into AAG polymorphic variants and their connection with various oncological and neurodegenerative diseases, including gene therapy and inhibitors, could enhance drug development. The repair of DNA damage is essential for preserving the integrity of genetic information and preventing the onset of numerous diseases. Gaining insight into how wild-type AAG and its polymorphic variants may relate to specific diseases may be useful for developing new treatments focused on modulating AAG activity. In this context, strategies aimed at both elevating AAG expression levels and creating inhibitors of its enzymatic activity may prove therapeutically beneficial. Elevating AAG expression levels may be advantageous in situations of excessive DNA damage, whereas developing inhibitors of AAG activity may aid in treating tumors with unregulated repair mechanisms. Investigating the impact of AAG polymorphisms on enzyme activity may assist in customizing treatment strategies to the distinct genetic profile of each patient, thereby enhancing the effectiveness of therapeutic interventions. Taken together, the dual role of AAG as a guardian of genomic stability and a potential driver of cancer underscores the need for a multidisciplinary approach.

## 7. Conclusions

The expression of enzyme polymorphic variants with modified activity may elevate the risk of cancer. Up to this point, only a limited number of studies have explored the role of AAG polymorphism in cancer progression. In this paper, we conducted a literature review as well as a bioinformatics analysis aimed at determining the impact of AAG polymorphic variants on its enzymatic characteristics. Over 370 missense SNPs were ranked based on their potential influence, with approximately 40% predicted to present a high-risk factor. The results obtained suggest a potential involvement of AAG polymorphic variants in the onset of oncological diseases. We compared the findings with data regarding the prevalence of specific SNPs in cancer samples. From the analysis, we identified 18 SNPs that combined the predicted negative impact and were detected in cancer samples. The majority of the identified mutations have not been functionally characterized previously and require experimental validation of the enzyme’s activity.

## Figures and Tables

**Table 1:** Amino acid mutations found in tumor samples according to COSMIC/cBioPortal/Portal.gdc.cancer.gov databases.

Cancers by Body Location/System	AAG Mutations
Breast	S47T, R100W, R120C, M151I
Digestive/Gastrointestinal	A5T, Q20H, R24Q, P25A, A28T, D36N, A37T, S47T, R60C, P64L, R72S, I74M, E89D, R100Q, R100W, L103V, R120H, E123V, E125K, Y127C, R145H, R147Q, G148S, M151I, P153L, G163D, G163S, R182Q, G189D, R197C, R201W, A205D, D211N, R212H, L214F, A226T, D239G, R246C, R246P, P254S, V264I, G265S, G281C
Endocrine	R194S, R197S, Q223P, P248A
Genitourinary	P112S, V158M, L180F, K202R, R261Q, H266N, F277L
Gynecologic	A5D, A5T, R17W, K21N, A38T, D50N, P65L, P68L, P94L, G114D, R118Q, M151I, P153Q, T192I, R207H, D211Y, E293K
Head and Neck	G265S
Hematologic/Blood	R17Q, T124I, S172R, H266Y
Musculoskeletal	R24Q
Neurologic	R118Q, P130S, M164I, D175N, L180F, V264A
Respiratory/ Thoracic	A52S, S77L, L180F, R246H, S282R
Skin	M22Q, S48L, R120H, M168I, R201Q, E213K, P248S, R261W, E269K, R272Q, L275I, R280W

**Table 2:** The dbSNP-derived AAG SNPs also found in tumor samples according to COSMIC, cBioPortal and National Cancer Institute databases.

№	SNP	Predicted Effect	Cancer Type	Conservation of Amino Acid Residue
1	P94L	High	Endometrioid carcinoma	Non conserved
2	R100W	High	Stomach carcinoma, breast carcinoma	Non conserved
3	R120C	High	Breast carcinoma	Non conserved
4	E125K	High	Upper aerodigestive tract carcinoma	Conserved
5	Y127C	High	Large intestine adenocarcinoma	Conserved
6	R145H	High	Stomach carcinoma	Conserved
7	P153L	High	Stomach carcinoma	Conserved
8	V158M	High	Kidney carcinoma, clear cell renal cell carcinoma	Conserved
9	G163S	High	Large intestine adenocarcinoma	Conserved
10	R182Q	High	Large intestine adenocarcinoma	Conserved
11	R197C	High	Esophagus adenocarcinoma	Non conserved
12	R207H	High	Endometrioid carcinoma, uterine adenomas and adenocarcinomas	Non conserved
13	R261Q	High	Urinary tract carcinoma	Non conserved
14	R261W	High	Malignant melanoma	Non conserved
15	E269K	High	Malignant melanoma	Non conserved
16	R280W	High	Cutaneous melanoma	Non conserved
17	G281C	High	Stomach adenocarcinoma	Non conserved
18	E293K	High	Cervix carcinoma, squamous cell carcinoma	Non conserved

## Data Availability

Data are available upon request to O.A.K. Tel. +7 (383) 363-5174, E-mail: kladova@1bio.ru.

## Supplementary Materials

The following supporting information can be downloaded at: https://www.mdpi.com/article/10.3390/ijms26157647/s1.
