# Supplementary material for: The Link Between Human Alkyladenine DNA Glycosylase and Cancer Development
Source: Int J Mol Sci. 2025 Aug 7;26(15):7647. doi: 10.3390/ijms26157647 (PMC12347255; doi:10.3390/ijms26157647)
Supplement: Supplementary file 1 [file ijms-26-07647-s001.zip › ijms-3753546-supplementary.pdf]

Supplementary material

The link between Human Alkyladenine DNA Glycosylase and Cancer Development

Kladova O.A.[1], Kuznetsova A.A.[1]

[1] Institute of Chemical Biology and Fundamental Medicine, Siberian Branch of Russian Academy of Sciences, Novosibirsk 630090, Russia;

* Correspondence: kladova@niboch.nsc.ru (O.A.K.); sandra-k@niboch.nsc.ru (A.A.K.)

Supplementary Table S1. The effect of amino acid substitutions in natural polymorphic variants of AAG.

D – indicates damaging predicted effect, N – neutral predicted effect.

The polymorphic variants for which experimental activity characterization was carried out are highlighted in blue.

№	SNP	SIFT	PolyPhen2	Mutation Taster	MetaRNN	PROVEAN	CADD	Result
1	C15F	D	N	N	N	N	N	Neutral
2	R16G	D	N	N	N	N	N	Neutral
3	R16Q	N	N	N	N	N	N	Neutral
4	R17G	D	N	N	N	N	D	Neutral
5	R17Q	D	N	N	N	N	D	Neutral
6	R17D	D	D	N	N	N	D	Medium
7	R17L	D	N	N	N	N	N	Neutral
8	M18V	N	N	D	N	N	N	Neutral
9	M18I	N	N	D	N	N	N	Neutral
10	K21N	D	D	N	N	N	D	Medium
11	K22Q*	N	N	N	N	N	D	Neutral
12	Q23K	N	N	N	N	N	D	Neutral
13	Q23H	D	D	D	N	N	D	Medium
14	R24G	D	N	D	N	N	D	Medium
15	R24Q	N	N	N	N	N	N	Neutral
16	P25N	N	N	N	N	N	N	Neutral
17	P25A	N	N	N	N	N	N	Neutral
18	A26D	N	N	N	N	N	N	Neutral
19	A26V	N	N	N	N	N	N	Neutral
20	A28G	D	N	N	N	N	N	Neutral
21	G29R	N	N	N	N	N	N	Neutral
22	H32N	N	N	N	N	N	N	Neutral
23	H32Y	D	N	N	N	N	N	Neutral
24	H32Q	N	N	N	N	N	N	Neutral
25	S33G	N	N	N	N	N	N	Neutral
26	S34L	N	N	N	N	N	N	Neutral
27	S34W	D	N	N	N	N	N	Neutral
28	D36N	N	N	N	N	N	N	Neutral
29	D36E	N	N	N	N	N	N	Neutral
30	A37N	N	N	N	N	N	N	Neutral
31	A37D	N	N	N	N	N	N	Neutral
32	A37V	N	N	N	N	N	N	Neutral
33	Q39H	D	N	N	N	N	N	Neutral
34	A40N	N	N	N	N	N	N	Neutral
35	A40D	N	N	N	N	N	N	Neutral
36	A40S	N	N	N	N	N	N	Neutral

37	A40V	N	N	N	N	N	N	Neutral
38	P41A	N	D	N	N	N	N	Neutral
39	P41S	N	D	N	N	N	N	Neutral
40	E43G	D	N	N	N	N	N	Neutral
41	E43K	N	N	N	N	N	N	Neutral
42	Q44E	N	N	N	N	N	N	Neutral
43	P45R	N	N	N	N	N	N	Neutral
44	S47N	N	N	N	N	N	N	Neutral
45	S47R	N	N	N	N	N	N	Neutral
46	S48L	N	N	N	N	N	N	Neutral
47	D50N	D	N	N	N	N	N	Neutral
48	D50H	N	N	N	N	N	N	Neutral
49	D50V	D	N	N	N	N	N	Neutral
50	A51S	N	N	N	N	N	N	Neutral
51	A52D	N	N	N	N	N	N	Neutral
52	A52V	N	N	N	N	N	N	Neutral
53	Q53R	N	N	N	N	N	N	Neutral
54	Q53L	N	N	N	N	N	N	Neutral
55	Q53H	N	N	N	N	N	N	Neutral
56	A54V	N	N	N	N	N	D	Neutral
57	P55S	N	N	N	N	N	N	Neutral
58	P55H	D	D	N	N	N	D	Medium
59	P55R	N	N	N	N	N	N	Neutral
60	C56S	N	N	N	N	N	N	Neutral
61	P57N	N	N	N	N	N	N	Neutral
62	R58W	D	N	N	N	N	N	Neutral
63	E59K	N	N	N	N	N	N	Neutral
64	R60S	N	N	N	N	N	N	Neutral
65	R60C	D	N	N	N	N	D	Neutral
66	R60H	N	N	N	N	N	N	Neutral
67	C61R	N	N	N	N	N	N	Neutral
68	C61G	N	N	N	N	N	N	Neutral
69	C61S	N	N	N	N	N	N	Neutral
70	G63E	D	N	N	N	N	N	Neutral
71	P64R	N	N	N	N	N	N	Neutral
72	P64L*	N	N	N	N	N	N	Neutral
73	P65A	D	N	N	N	N	N	Neutral
74	P65R	D	N	N	N	N	D	Neutral
75	T66S	N	N	N	N	N	N	Neutral
76	T67I	D	N	N	N	N	N	Neutral
77	G69R	D	D	N	N	N	D	Medium
78	G69D	D	D	N	N	N	D	Medium
79	P70A	N	N	N	N	N	N	Neutral
80	P70S	N	N	N	N	N	N	Neutral
81	P70R	N	D	N	N	N	D	Neutral
82	P70L	N	N	N	N	N	N	Neutral

83	Y71H*	N	N	N	N	N	N	Neutral
84	R72G	D	N	N	N	N	N	Neutral
85	R72C	D	D	N	N	N	D	Medium
86	R72H	D	N	N	N	N	D	Medium
87	S73N	D	D	D	N	N	D	Medium
88	I74V	N	N	N	N	N	N	Neutral
89	Y75H	D	D	D	N	N	D	Medium
90	Y75S	D	D	D	D	D	D	High
91	Y75C	D	D	D	D	D	D	High
92	S77L	N	N	N	N	N	N	Neutral
93	P79L	N	N	N	N	N	N	Neutral
94	K80E	N	N	N	N	N	N	Neutral
95	H82Y	N	N	N	N	N	N	Neutral
96	L83R	N	N	N	N	N	N	Neutral
97	T84A	N	N	N	N	N	N	Neutral
98	R85G	D	D	N	D	D	D	High
99	R85Q	N	N	N	D	D	D	Medium
100	R85L	D	N	N	N	N	D	Medium
101	L88M	N	N	N	N	N	N	Neutral
102	L88F	N	N	N	N	N	N	Neutral
103	L88S	N	N	N	N	N	N	Neutral
104	F91L	D	D	D	D	D	D	High
105	D92N	N	N	N	N	N	N	Neutral
106	D92H	D	D	N	D	D	D	High
107	D92A	N	D	D	D	D	D	High
108	Q93K	N	N	D	N	N	D	Neutral
109	Q93D	N	D	D	D	D	D	High
110	Q93R*	N	N	D	N	N	D	Neutral
111	P94S	N	D	D	D	D	D	High
112	P94L	D	D	D	D	D	D	High
113	A95N	N	N	N	N	N	D	Neutral
114	A95V	D	N	N	N	N	D	Neutral
115	P97L	N	N	N	N	N	D	Neutral
116	A99V	D	D	D	D	D	D	High
117	R100W	D	D	N	D	D	D	High
118	R100D	D	D	N	D	D	D	High
119	R100L	D	N	N	D	D	D	Medium
120	R100Q	N	N	N	N	N	N	Medium
121	A101S	N	D	D	N	N	D	Medium
122	A102E	D	D	N	N	N	D	Medium
123	F102V	D	D	D	D	D	D	High
124	L103D	D	D	D	D	D	D	High
125	G104R	D	N	D	D	D	D	High
126	Q105H	N	N	D	N	N	D	Medium
127	V108G	D	D	D	D	D	D	High
128	V108I	D	D	D	N	N	D	High

129	R109G	D	D	D	D	D	D	High
130	R109Q	D	D	D	N	D	D	High
131	R110Q	N	N	N	N	N	D	High
132	L111W	N	D	D	N	N	D	Medium
133	L111V	D	D	D	D	D	D	High
134	N113F	N	N	N	N	N	D	Neutral
135	T115S	N	D	D	D	D	D	High
136	L117R	D	D	D	D	D	D	High
137	L117F	D	D	D	N	D	D	High
138	R118Q	N	D	D	N	N	D	Medium
139	G119D	D	D	D	D	D	D	High
140	R120C*	N	D	D	D	D	D	High
141	R120H	D	D	D	N	D	D	High
142	I121V	D	N	D	N	N	N	Neutral
143	I121N	D	D	D	D	D	D	High
144	V122M	D	D	D	D	D	D	High
145	V122A	D	D	D	D	D	D	High
146	E123K	D	D	D	D	D	D	High
147	T124N	D	D	D	D	D	D	High
148	E125K	D	D	D	D	D	D	High
149	Y127C	D	D	D	D	D	D	High
150	L128M	N	D	D	N	N	D	Medium
151	L128V	N	N	D	N	N	N	Neutral
152	G129W	D	D	D	D	D	D	High
153	P130L	N	N	D	N	N	D	Neutral
154	D132N	D	D	D	D	D	D	High
155	D132G	D	D	D	D	D	D	High
156	E133K	N	N	D	N	N	D	Neutral
157	A135N	D	D	D	N	N	D	Medium
158	H136D	D	D	D	D	D	D	High
159	H136R	D	D	D	D	D	D	High
160	S137N	N	D	D	N	N	D	Medium
161	S137L	D	D	D	D	D	D	High
162	R138K	N	N	N	N	N	D	Neutral
163	R138M	N	D	D	N	N	D	Medium
164	G140S	D	D	D	D	D	D	High
165	G140C	D	D	D	D	D	D	High
166	G140D	D	D	D	D	D	D	High
167	R141W	D	D	D	D	D	D	High
168	R141Q*	D	D	D	N	N	D	Medium
169	Q142R	N	N	D	N	N	D	Neutral
170	Q142H	N	D	D	N	N	D	Medium
171	T143D	D	D	D	D	D	D	High
172	T143N	D	D	D	D	D	D	High
173	T143S	D	N	D	D	D	D	High
174	T143I	D	D	D	D	D	D	High

175	P144S	N	N	N	N	N	D	Neutral
176	P144L	N	N	D	D	D	D	Medium
177	R145S	D	D	D	D	D	D	High
178	R145G	D	N	D	D	D	D	High
179	R145C	D	N	D	D	D	D	High
180	R145H	D	D	D	D	D	D	High
181	N146H	D	D	D	D	D	D	High
182	N146D	D	D	D	D	D	D	High
183	N146N	N	D	D	D	D	D	High
184	N146S	D	N	D	D	D	D	High
185	R147G	N	N	N	D	D	D	Medium
186	R147Q	N	N	N	N	N	D	Neutral
187	G148S	N	N	D	N	N	D	Neutral
188	G148R	N	D	D	N	N	D	Medium
189	G148V	N	D	D	N	N	D	Medium
190	M151N	D	D	D	D	D	D	High
191	P153S	D	D	D	D	D	D	High
192	P153R	D	D	D	D	D	D	High
193	P153L	D	D	D	D	D	D	High
194	G154E	D	D	D	D	D	D	High
195	T155A	D	D	D	D	D	D	High
196	T155N	D	D	D	D	D	D	High
197	T155I	N	D	D	D	D	D	High
198	L156D	N	D	D	D	D	D	High
199	V158M	D	D	D	D	D	D	High
200	V158A	D	D	D	D	D	D	High
201	Y159S	D	D	D	D	D	D	High
202	I161N	N	D	D	N	N	D	Medium
203	Y162C	D	D	D	D	D	D	High
204	G163S	D	D	D	D	D	D	High
205	G163A	D	D	D	D	D	D	High
206	G163V	D	D	D	D	D	D	High
207	M164V	D	N	D	D	D	D	High
208	M164L	N	N	D	N	N	D	Neutral
209	M164N	D	N	D	D	D	D	High
210	M164R	D	N	D	D	D	D	High
211	Y165C	D	D	D	D	D	D	High
212	M168V	N	N	N	N	N	D	Neutral
213	M168I	N	N	D	N	N	D	Neutral
214	I170V	N	N	N	N	N	N	Neutral
215	I170M	D	D	N	D	D	D	High
216	S172C	D	D	D	D	D	D	High
217	G174R	D	D	D	D	D	D	High
218	G174A	D	D	D	D	D	D	High
219	D175N	N	N	D	D	D	D	Medium
220	G176R	D	D	D	D	D	D	High

221	G176W	D	D	D	D	D	D	High
222	G176E	D	D	D	D	D	D	High
223	A177D	D	D	D	D	D	D	High
224	A177S	N	D	D	N	N	D	Medium
225	C178R	D	D	D	D	D	D	High
226	C178Y	D	D	D	D	D	D	High
227	C178S	N	D	D	N	N	D	Medium
228	C178W	D	D	D	D	D	D	High
229	V179I	D	D	D	N	N	D	Medium
230	L180V	D	D	D	D	D	D	High
231	R182G	D	D	D	D	D	D	High
232	R182Q	D	D	D	D	D	D	High
233	L187D	D	D	D	D	D	D	High
234	L187Q	D	D	D	D	D	D	High
235	E188K	N	N	N	N	N	D	Neutral
236	G189S	D	D	D	D	D	D	High
237	G189V	D	D	D	D	D	D	High
238	L190D	D	D	D	D	D	D	High
239	E191Q	D	D	D	N	N	D	Medium
240	M193V	D	D	D	D	D	D	High
241	M193N	D	D	D	D	D	D	High
242	R194C	D	D	D	D	D	D	High
243	R194H	D	D	D	D	D	D	High
244	R197C	D	D	D	D	D	D	High
245	R197H	D	D	D	D	D	D	High
246	R197L	D	D	D	D	D	D	High
247	S198G	N	N	N	N	N	N	Neutral
248	S198R	N	N	N	N	N	N	Neutral
249	T199D	N	N	N	N	N	N	Neutral
250	T199N	N	N	N	N	N	N	Neutral
251	L200R	N	N	N	N	N	N	Neutral
252	R201G	D	N	N	D	D	D	Medium
253	R201W	D	N	N	D	D	D	Medium
254	R201Q	N	N	N	N	N	D	Medium
255	G203S	N	N	N	N	N	D	Neutral
256	T204D	N	N	N	N	N	N	Neutral
257	T204S	N	N	N	N	N	N	Neutral
258	A205N	N	N	N	N	N	N	Neutral
259	A205D	N	D	N	N	N	N	Neutral
260	R207C	D	D	N	D	D	D	High
261	R207H	D	D	N	D	D	D	High
262	V208G	N	N	D	N	N	N	Neutral
263	L209F	N	N	D	N	N	D	Neutral
264	K210E	D	D	D	D	D	D	High
265	K210N	D	D	D	D	D	D	High
266	D211H	D	D	D	D	D	D	High

267	R212C	D	D	D	D	D	D	High
268	R212H	N	N	N	N	N	D	Neutral
269	E213K	N	N	D	D	D	D	Medium
270	E213G	N	D	D	D	D	D	High
271	C215Y	D	D	D	D	D	D	High
272	G217D	D	D	D	D	D	D	High
273	P218H	D	D	D	D	D	D	High
274	S219D	D	D	D	D	D	D	High
275	S219F	D	D	D	D	D	D	High
276	K220Q	D	D	D	D	D	D	High
277	K220R	D	D	D	D	D	D	High
278	L221R	D	D	D	D	D	D	High
279	C222Y	D	D	D	D	D	D	High
280	A224V	D	D	D	D	D	D	High
281	K229R	N	N	D	N	N	D	Neutral
282	S230N	N	N	D	N	N	D	Neutral
283	F231I	N	N	D	N	N	D	Neutral
284	D232N	N	D	D	N	N	D	Medium
285	Q233E	N	D	D	N	N	D	Medium
286	Q233H	N	D	D	N	N	D	Medium
287	D235N	N	D	D	D	D	D	High
288	L236V	D	D	D	N	N	D	Medium
289	A237N	N	N	D	N	N	D	Neutral
290	A237D	N	N	D	N	N	D	Neutral
291	A237V	N	N	D	N	N	D	Neutral
292	Q238K	N	N	N	N	N	N	Neutral
293	Q238H	N	N	N	N	N	N	Neutral
294	D239N	N	D	D	N	N	D	Medium
295	D239Y	D	D	D	D	D	D	High
296	D239E	N	N	N	N	N	N	Neutral
297	E240D	N	N	N	N	N	N	Neutral
298	A241N	N	N	N	N	N	D	Neutral
299	V242I	N	N	N	N	N	N	Neutral
300	V242L	N	N	N	N	N	N	Neutral
301	W243R	D	D	D	D	D	D	High
302	W243C	D	D	D	D	D	D	High
303	E245K	D	D	D	D	D	D	High
304	E245Q	D	D	D	N	N	D	Medium
305	E245A	D	D	D	D	D	D	High
306	R246C	N	N	N	D	D	N	Neutral
307	R246H	N	N	N	N	N	N	Neutral
308	G247R	D	N	N	D	D	D	Medium
309	L249D	N	N	N	N	N	N	Neutral
310	E250D	N	N	N	N	N	N	Neutral
311	P251S	N	N	N	N	N	N	Neutral
312	E253K	N	N	N	N	N	N	Neutral

313	P254Q	N	N	N	N	N	N	Neutral
314	P254L	N	N	N	N	N	N	Neutral
315	A255G	N	N	N	N	N	N	Neutral
316	V256I	N	N	D	N	N	D	Neutral
317	V256L	D	N	D	N	N	D	Medium
318	A258V*	N	N	N	N	N	D	Neutral
319	A259N	N	D	D	N	N	D	Medium
320	A259G	N	N	D	D	D	D	Medium
321	R261W	D	D	D	D	D	D	High
322	R261Q	D	D	D	D	D	D	High
323	R261D	D	D	D	D	D	D	High
324	V262L	D	N	N	N	N	D	Neutral
325	V262A	D	N	D	D	D	D	High
326	V262G	D	D	D	D	D	D	High
327	G263D	D	D	D	D	D	D	High
328	G263A	D	D	D	D	D	D	High
329	V264I	N	N	N	N	N	N	Neutral
330	V264L	N	N	N	N	N	N	Neutral
331	G265S	N	N	N	N	N	D	Neutral
332	A267N	N	N	N	N	N	N	Neutral
333	G268R	N	D	D	D	D	D	High
334	G268V	N	D	D	D	D	D	High
335	E269K	N	D	D	D	D	D	High
336	A271N	N	N	D	N	N	D	Neutral
337	R272W	D	D	N	D	D	D	High
338	R272Q	N	N	N	N	N	N	Neutral
339	K273N	D	D	D	D	D	D	High
340	P274N	N	D	D	D	D	D	High
341	P274R	D	D	D	D	D	D	High
342	P274L	N	N	D	D	D	D	Medium
343	L275V	D	D	D	D	D	D	High
344	L275F	N	D	D	D	D	D	High
345	L275D	D	D	D	D	D	D	High
346	R276G	D	D	D	D	D	D	High
347	R276C	D	D	D	D	D	D	High
348	R276H	D	D	D	D	D	D	High
349	Y278C	D	D	D	D	D	D	High
350	V279I	N	N	N	N	N	N	Neutral
351	R280W	D	D	N	D	D	D	High
352	R280Q	N	N	N	N	N	N	Neutral
353	R280D	N	D	N	D	D	D	Medium
354	G281S	D	D	D	D	D	D	High
355	G281C	D	D	D	D	D	D	High
356	P283S	N	N	D	D	D	D	Medium
357	W284R	N	D	D	D	D	D	High
358	V285I	N	D	D	N	N	D	Medium

359	S286G	D	D	D	D	D	D	High
360	V288I	N	N	D	N	N	D	Neutral
361	V288A	D	N	D	N	N	D	Medium
362	D289N	N	N	D	D	D	D	Medium
363	R290K	N	N	N	N	N	N	Neutral
364	V291M	N	N	N	N	N	D	Neutral
365	A292N	N	N	N	N	N	D	Neutral
366	A292V	N	N	N	N	N	D	Neutral
367	E293K	D	D	D	D	D	D	High
368	E293G	D	D	D	D	D	D	High
369	Q294E	D	N	N	N	N	N	Neutral
370	D295N	N	N	N	N	N	N	Neutral
371	D295Y	D	N	N	N	N	N	Neutral
372	T296I	N	N	N	N	N	N	Neutral
373	Q297K	D	N	N	N	N	N	Neutral
374	Q297E	N	N	N	N	N	N	Neutral
375	Q297R	D	N	N	N	N	N	Neutral
376	A298S*	N	N	N	N	N	N	Neutral

* SNP variants experimentally characterized in Adhikari, S.; Chetram, M.A.; Woodrick, J.; Mitra, P.S.; Manthena, P. V.; Khatkar, P.; Dakshanamurthy, S.; Dixon, M.; Karmahapatra, S.K.; Nuthalapati, N.K.; et al. Germ Line Variants of Human N-Methylpurine DNA Glycosylase Show Impaired DNA Repair Ac-tivity and Facilitate 1,N6-Ethenoadenine-Induced Mutations. J Biol Chem 2015, 290, 4966, doi:10.1074/JBC.M114.627000.

Supplementary Table S2. Occurrence of AAG mutations in tumors.

№		COSMIC<br>https://cancer.sanger.ac.uk/cosmic (accessed on 10 February 2025)	Cbioportal<br>https://www.cbioportal.org/ (accessed on 10 February 2025)	Portal.gdc.cancer.gov<br>https://portal.gdc.cancer.gov/ (accessed on 10 February 2025)
**1**	**A5D**	Endometrioid carcinoma	Endometrioid carcinoma	Uterine adenocarcinoma
**2**	**A5T**	Large intestine adenoma	Endometrioid carcinoma	Uterine adenocarcinoma
**3**	**R17Q**			Acute lymphoblastic leukemia
**4**	**R17W**	Endometrioid carcinoma	Endometrioid carcinoma	Uterine adenocarcinomas
**5**	**Q20H**			Stomach adenomas and adenocarcinomas
**6**	**K21N**		Uterine carcinosarcoma	Uterus, NOS complex mixed and stromal neoplasms
**7**	**K22Q**	Malignant melanoma		
**8**	**R24Q**	Rhabdomyosarcoma, large intestine adenocarcinoma		
**9**	**P25A**	Biliary tract adenocarcinoma		
**10**	**A28T**	Stomach carcinoma		
**11**	**D36N**	Stomach adenocarcinoma	Intestinal type stomach adenocarcinoma	Stomach adenomas and adenocarcinomas
**12**	**A37T**	Colon adenocarcinoma		
**13**	**A38T**	Endometrioid carcinoma	Uterine serous carcinoma	Uterine adenomas and adenocarcinomas
**14**	**S47T**	Breast carcinoma, large intestine adenocarcinoma	Breast invasive ductal carcinoma	Breast adenomas and adenocarcinomas
**15**	**S48L**	Malignant melanoma	Cutaneous melanoma	Skin nevi and melanomas
**16**	**D50N**	Endometrioid carcinoma	Endometrioid carcinoma	Uterine adenomas and adenocarcinomas
**17**	**A52S**	Non small cell carcinoma		
**18**	**R60C**	Large intestine adenocarcinoma	Colon adenocarcinoma	

**19**	**P64L**	Upper aerodigestive tract carcinoma		
**20**	**P65L**	Ovary carcinoma		
**21**	**P68L**	Endometrioid carcinoma	Endometrioid carcinoma	
**22**	**R72S**	Large intestine adenocarcinoma		
**23**	**I74M**	Hepatocellular carcinoma	Hepatocellular carcinoma	Liver and intrahepatic bile ducts adenomas and adenocarcinomas
**24**	**S77L**	Lung adenocarcinoma		
**25**	**E89D**	Stomach carcinoma		
**26**	**P94L**	Endometrioid carcinoma	Endometrioid carcinoma	Uterine adenomas and adenocarcinomas
**27**	**R100Q**	Large intestine adenocarcinoma	Colon adenocarcinoma	Colon adenomas and adenocarcinomas
**28**	**R100 W**	Stomach carcinoma, breast carcinoma		
**29**	**L103V**	Stomach carcinoid-endocrine tumour		
**30**	**P112S**	Urinary tract carcinoma	Bladder urothelial carcinoma	Bladder adenomas and adenocarcinomas
**31**	**G114D**	Ovary carcinoma	Serous ovarian cancer	Ovary adenomas and adenocarcinomas
**32**	**R118Q**	Endometrioid carcinoma, astrocytoma grade IV	Glioblastoma multiforme, uterine endometrioid carcinoma	
**33**	**R120C**	Breast carcinoma	Breast invasive ductal carcinoma	
**34**	**R120H**	Malignant melanoma, large intestine adenocarcinoma		
**35**	**E123V**	Hepatocellular carcinoma		
**36**	**T124I**	Acute leukaemia of ambiguous lineage		
**37**	**E125K**	Upper aerodigestive tract carcinoma		
**38**	**Y127C**	Large intestine adenocarcinoma		

39	**P130S**		Astrocytoma	Brain Gliomas
40	**R145H**	Stomach carcinoma	Stomach adenocarcinoma	Stomach adenomas and adenocarcinomas
41	**R147Q**	Large intestine adenocarcinoma		
42	**G148S**	Oesophagus adenocarcinoma		
43	**M151I**			Breast, colon, ovary, rectum adenomas and adenocarcinomas
44	**P153L**	Stomach carcinoma		
45	**P153Q**			Uterine adenomas and adenocarcinomas
46	**V158 M**	Kidney carcinoma, clear cell renal cell carcinoma	Renal clear cell carcinoma	Kidney adenomas and adenocarcinomas
47	**G163D**	Large intestine adenocarcinoma		
48	**G163S**	Large intestine adenocarcinoma		
49	**M164I**	Glioma, astrocytoma grade IV		
50	**M168I**	Malignant melanoma		
51	**S172R**			Hematopoietic and reticuloendothelial systems, lymphoid leukemias
52	**D175N**	Meninges meningioma		
53	**L180F**			Brain, bronchus and lung, kidney pancreas adenomas and adenocarcinomas, gliomas
54	**R182Q**	Large intestine adenocarcinoma		
55	**G189D**		Esophageal adenocarcinoma	Esophagus stomach adenomas and adenocarcinomas
56	**T192I**		Endometrioid carcinoma	Uterine adenomas and adenocarcinomas

**57**	**R194S**	Thyroid carcinoma		
**58**	**R197C**	Oesophagus adenocarcinoma		
**59**	**R197S**	Thyroid carcinoma		
**60**	**R201Q**	Skin carcinoma, basal cell carcinoma		
**61**	**R201 W**	Large intestine adenocarcinoma		
**62**	**K202R**	Urinary tract carcinoma	Bladder urothelial carcinoma	Bladder adenomas and adenocarcinomas
**63**	**A205D**	Stomach adenocarcinoma	Diffuse type stomach adenocarcinoma	Stomach adenomas and adenocarcinomas
**64**	**R207H**		Endometrioid carcinoma	Uterine adenomas and adenocarcinomas
**65**	**D211N**		Esophageal squamous cell carcinoma	Esophagus stomach adenomas and adenocarcinomas
**66**	**D211Y**	Ovary carcinoma	Serous ovarian cancer	Ovary cystic, mucinous and serous neoplasms
**67**	**R212H**	Stomach adenocarcinoma	Stomach adenocarcinoma	Stomach adenomas and adenocarcinomas
**68**	**E213K**	Malignant melanoma		
**69**	**L214F**	Large intestine adenocarcinoma	Colon adenocarcinoma	Colon adenomas and adenocarcinomas
**70**	**Q223P**	Thyroid neoplasm		
**71**	**A226T**	Large intestine adenocarcinoma		
**72**	**D239G**	Liver carcinoma		
**73**	**R246C**	Large intestine adenocarcinoma	Mucinous adenocarcinoma of the colon and rectum	Colon adenomas and adenocarcinomas
**74**	**R246H**	Lung carcinoid-endocrine tumour		
**75**	**R246P**	Oesophagus carcinoma, squamous cell carcinoma		
**76**	**P248A**	Adrenal gland pheochromocytoma		

**77**	**P248S**	Skin carcinoma, basal cell carcinoma		
**78**	**P254S**	Large intestine adenocarcinoma	Mucinous adenocarcinoma of the colon and rectum	Colon adenomas and adenocarcinomas
**79**	**R261Q**	Urinary tract carcinoma	Bladder urothelial carcinoma	Bladder adenomas and adenocarcinomas
**80**	**R261 W**	Malignant melanoma		
**81**	**V264A**	Glioma, astrocytoma grade IV	Glioblastoma multiforme	Gliomas
**82**	**V264I**	Stomach carcinoma		
**83**	**G265S**	Upper aerodigestive tract carcinoma, nasopharyngeal carcinoma		
**84**	**H266N**	Kidney carcinoma, papillary renal cell carcinoma	Papillary renal cell carcinoma	Kidney adenomas and adenocarcinomas
**85**	**H266Y**	Hodgkin lymphoma		
**86**	**E269K**	Malignant melanoma		
**87**	**R272Q**	Malignant melanoma		
**88**	**L275I**	Malignant melanoma	Cutaneous melanoma	
**89**	**F277L**	Prostate carcinoma		
**90**	**R280 W**		Cutaneous melanoma	Skin nevi and melanomas
**91**	**G281C**	Stomach adenocarcinoma	Diffuse type stomach adenocarcinoma	
**92**	**S282R**	Lung carcinoma, squamous cell carcinoma		
**93**	**E293K**	Cervix carcinoma, squamous cell carcinoma	Mucinous carcinoma	Cervix uteri adenomas and adenocarcinomas
